# Crystal structure of the monocupin ring-cleaving dioxygenase 5-nitrosalicylate 1,2-dioxygenase from *Bradyrhizobium* sp.

**DOI:** 10.1107/S2059798323004199

**Published:** 2023-06-16

**Authors:** Erik Eppinger, Andreas Stolz, Marta Ferraroni

**Affiliations:** a Institut für Mikrobiologie, Universität Stuttgart, Allmandring 31, 70569 Stuttgart, Germany; b Dipartimento di Chimica ‘Ugo Schiff’, Università di Firenze, Via della Lastruccia 3, 50019 Sesto Fiorentino (FI), Italy; Lund University, Sweden

**Keywords:** dioxygenases, 5-nitrosalicylate 1,2-dioxygenase, *Bradyrhizobium* sp JS329, X-ray crystallography, cupins, substrate specificity, aromatic catabolism

## Abstract

5-Nitrosalicylate 1,2-dioxygenase (5NSDO) is an iron(II)-dependent dioxygenase involved in the aerobic degradation of 5-nitroanthranilic acid by the bacterium *Bradyrhizobium* sp. It catalyzes the opening of the 5-nitrosalicylate aromatic ring, a key step in the degradation pathway. Besides 5-nitrosalicylate, the enzyme is also active towards 5-chlorosalicylate. The X-ray crystallographic structure of the enzyme was solved at 2.1 Å resolution by molecular replacement using a model from the AI program *AlphaFold*. The enzyme crystallized in the monoclinic space group *P*2_1_, with unit-cell parameters *a* = 50.42, *b* = 143.17, *c* = 60.07 Å, β = 107.3°. 5NSDO belongs to the third class of ring-cleaving dioxygenases. Members of this family convert *para*-diols or hydroxylated aromatic carboxylic acids and belong to the cupin superfamily, which is one of the most functionally diverse protein classes and is named on the basis of a conserved β-barrel fold. 5NSDO is a tetramer composed of four identical subunits, each folded as a monocupin domain. The iron(II) ion in the enzyme active site is coordinated by His96, His98 and His136 and three water molecules with a distorted octahedral geometry. The residues in the active site are poorly conserved compared with other dioxygenases of the third class, such as gentisate 1,2-dioxygenase and salicylate 1,2-dioxygenase. Comparison with these other representatives of the same class and docking of the substrate into the active site of 5NSDO allowed the identification of residues which are crucial for the catalytic mechanism and enzyme selectivity.

## Abstract 2

The crystal structure of the monocupin 5-nitrosalicylate 1,2-dioxygenase, an iron(II)-dependent ring-cleaving dioxygenase, from *Bradyrhizobium* sp. was determined by molecular replacement using a theoretical model obtained by *AlphaFold*2. Comparison with structures of other members of the same class and docking of the substrate allowed identification of the residues responsible for its very unusual enzyme selectivity.

## 1. Introduction

Nitroaromatic compounds, which are widely used as dyes, pesticides, synthetic intermediates and explosives, are particu­larly toxic and recalcitrant to degradation (Ju & Parales, 2010 ▸).

5-Nitrosalicylate dioxygenase (EC 1.13.11.64; 5NSDO), isolated from the soil bacterium *Bradyrhizobium* sp. JS329, is an enzyme involved in the degradation of 5-nitroanthranilic acid, which is produced biologically for an unknown purpose by *Streptomyces scabies* and also industrially in the synthesis of nitroaromatic products and dyes (Qu & Spain, 2010 ▸, 2011 ▸).

The degradation pathway is initiated by hydrolytic removal of the amino group catalyzed by an aminohydrolase to form 5-nitrosalicylate, which is then oxidized by 5NSDO, which cleaves the aromatic ring with the production of 4-nitro-6-oxohepta-2,4-dienedioic acid. The resulting ring-fission product undergoes spontaneous lactonization accompanied by removal of the nitro group as nitrite. This spontaneous removal of the nitro group is unusual. In the bio­degradation of synthetic nitroaromatic compounds the nitro group is generally eliminated in the initial step by an oxygenase or a reductase (Ju & Parales, 2010 ▸). Lactone ring opening is then catalyzed by a lactone hydrolase to form maleyl­pyruvate, which is in turn hydrolyzed to produce intermediates of central metabolism.

Many aerobic degradation pathways of aromatic compounds by microorganisms converge to catecholic substrates which can undergo an *ortho* cleavage catalyzed by intradiol dioxygenases or a *meta* cleavage catalyzed by extradiol dioxygenases (Vaillancourt *et al.*, 2006 ▸). However, in several bacterial degradation pathways the intermediates are converted to *para*-diols or hydroxylated aromatic carboxylic acids. The oxidation of these noncatecholic substrates is catalyzed by a third class of less studied dioxygenases which cleave the ring between one hydroxyl substituent and the adjacent C atom, specifically that carrying the carboxylate in hydroxylated carboxylic acids (Fetzner, 2012 ▸).

This class is part of the vast cupin superfamily, which is one of the most functionally diverse protein classes and is named on the basis of a conserved β-barrel fold (Dunwell *et al.*, 2004 ▸). Members of this superfamily all share a common architecture composed of a motif of 6–8 antiparallel β-strands located within a conserved β-barrel structure. They are quite functionally diverse and include non­enzymatic proteins such as plant seed storage proteins, transcription regulators and stress-related proteins, and a wide variety of enzymes such as epimerases, isomerases, carboxylases and many oxygenases. Members of the cupin dioxygenase family either comprise a single cupin domain (monocupins) or have a duplicated domain structure (bi­cupins) (Fetzner, 2012 ▸).

5NSDO is a nonheme iron(II)- and oxygen-dependent enzyme that belongs to the third class of ring-cleaving dioxygenases as it cleaves the aromatic ring of 5-nitrosalicylate, an *ortho*-hydroxy-substituted carboxylic acid. The enzyme also catalyzes the oxidation of 5-chlorosalicylate, while it is not active towards gentisate, salicylate, 5-hydroxyanthranilate, 4-nitrocatechol or 4-chlorocatechol (Qu & Spain, 2010 ▸).

Among the biochemically characterized cupin ring-cleaving dioxygenases, salicylate, 1-hydroxy-2-naphthoate and gentisate 1,2-dioxygenases are the most closely related to 5NSDO. Gentisate 1,2-dioxygenases (GDOs) generally have a narrow substrate specificity (Harpel & Lipscomb, 1990 ▸), whereas salicylate 1,2-dioxygenase (SDO) appears to be more versatile because in addition to gentisate it also converts a wide range of substituted salicylates and 1-hydroxy-2-naphthoate (Hintner *et al.*, 2001 ▸, 2004 ▸). Gentisate and salicylate 1,2-dioxygenases have already been structurally characterized. X-ray structures of GDO from *Escherichia coli* O157:H7 (Adams *et al.*, 2006 ▸) and *Silicibacter pomeroyi* DSS-3 (Chen *et al.*, 2008 ▸) and of SDO from *Pseudaminobacter salicylatoxidans* BN12 (Matera *et al.*, 2008 ▸) have been solved. Furthermore, the X-ray structures of a number of SDO mutants (Ferraroni, Steimer *et al.*, 2012 ▸) and of complexes of wild-type SDO and of the G106A mutant (Ferraroni, Matera *et al.*, 2012 ▸; Ferraroni *et al.*, 2013 ▸) with substrates have been determined.

In these enzymes the active site is centered on the iron(II) ion, which is generally coordinated to three histidines. Other more distantly related dioxygenases of the cupin class such as 3-hydroxyanthranilate 3,4-dioxygenase have a different coordination at the iron ion composed of two histidines and one glutamate, resembling the 2-His-1-carboxylate facial triad of extradiol dioxygenases (Fetzner, 2012 ▸).

The catalytic mechanism proposed for GDOs and SDO, which is very similar to that thoroughly investigated for the extradiol dioxygenases (Lipscomb, 2008 ▸), starts with bidentate coordination of the substrate via its hydroxyl and carboxylate groups to the iron(II) ion, which is activated for binding the oxygen. Consequently, a semiquinone–iron(II)–superoxide species is formed, resulting in polarization of electron density away from the aromatic ring of the substrate towards the iron-bound O_2_. The metal center is presumed to act as a conduit for single-electron transfer from the metal-bound substrate to O_2_. Recombination of the semiquinone superoxo biradical species by attack of O_2_ at the electron-deficient C1 of the bound substrate forms an alkylperoxo intermediate. Subsequent rearrangement and O—O bond cleavage gives a seven-membered lactone intermediate and an iron(II)-bound hydroxide ion, which hydrolyzes the lactone to yield the product. Roles for some acid–base residues in the mechanism have been proposed. They should act as bases to deprotonate the substrate when it binds to the metal ion, later assisting as a proton donor in formation of the superoxide radical and in rearrangement of the alkylperoxo intermediate (Harpel & Lipscomb, 1990 ▸; Adams *et al.*, 2006 ▸; Chen *et al.*, 2008 ▸; Matera *et al.*, 2008 ▸; Eppinger *et al.*, 2015 ▸). The peculiar substrate selectivity of 5NSDO prompted us to investigate this enzyme more thoroughly. Here, we report the X-ray crystallographic structure of 5NSDO from *Bradyrhizobium* sp., which allowed us to shed some light on the molecular determinants of its unusual substrate selectivity.

## 2. Materials and methods

### 2.1. Expression and purification of 5NSDO

Plasmid pJS804 was used as a source of the gene (*naaB*) coding for 5NSDO from *Bradyrhizobium* sp. JS329 (Qu & Spain, 2011 ▸).

5NSDO was produced using *E. coli* Rosetta (DE3) pLysS (pJS804) cells. The cells were grown overnight at 37°C in 20 ml LB medium with ampicillin (100 µg ml^−1^) and chloram­phenicol (25 µg ml^−1^). This preculture was used to inoculate 2 l Erlenmeyer flasks containing the same medium (500 ml) to an initial optical density (OD_600 nm_) of about 0.1. The cultures were incubated at 37°C and 100 rev min^−1^ until an OD_600 nm_ of 0.6–0.7 was reached. Subsequently, the bacterial culture was transferred to room temperature (23°C) and expression of 5NSDO was induced by adding isopropyl β-d-1-thiogalactopyranoside (IPTG) to a final concentration of 0.1 m*M*. After 5 h, the cells were harvested by centrifugation (8000 rev min^−1^, 15 min, 4°C) and washed twice with ice-cold 20 m*M* potassium phosphate buffer pH 7.

The 5NSDO was purified at room temperature using an ÄKTA FPLC system (GE Healthcare). The washed *E. coli* Rosetta(DE3) pLysS (pJS804) cells were resuspended to an OD_600 nm_ of 200 in 20 m*M* potassium phosphate buffer pH 7 and disrupted using a French press (Aminco, Silver Springs, Maryland, USA) at 80 MPa. Intact cells and cell debris were removed by ultracentrifugation (82 000*g*, 4°C, 1 h). The protein content of the cell extracts was determined by the method of Bradford (1976 ▸) using bovine serum albumin as the standard. The crude extract was loaded onto a HiPrep 26/10 column (53 ml bed volume, 5 kDa cutoff; GE Healthcare) equilibrated with 300 ml 20 m*M* bis-Tris–HCl buffer pH 7.2. The proteins were eluted with 20 m*M* bis-Tris–HCl buffer pH 7.2 at a flow rate of 8 ml min^−1^. Protein-containing fractions (2 ml each) were collected and loaded onto an anion-exchange column (HiTrap Q FF, 5 ml bed volume; GE Healthcare). The protein was eluted with a 40 ml linear gradient from 20 m*M* bis-Tris–HCl pH 7.2 to 20 m*M* bis-Tris–HCl pH 7.2, 1 *M* NaCl at a flow rate of 1 ml min^−1^. 1 ml fractions were collected and analyzed by SDS–PAGE. 5NSDO eluted at a concentration of about 150 m*M* NaCl. The pooled 5NSDO fractions were combined and concentrated by ultrafiltration (Vivaspin 500, 10 kDa exclusion volume; Vivascience AG) to a final volume of 2 ml. The concentrated 5NSDO fraction was subsequently polished using a Superdex 200 Prep-Grade 16/60 column (bed volume 102 ml; GE Healthcare) at a flow rate of 1 ml min^−1^ using 20 m*M* potassium phosphate buffer pH 7.0, 150 m*M* NaCl as the elution buffer. The 5NSDO fractions (1 ml each) were combined and concentrated by ultrafiltration (Vivaspin 500, 10 kDa exclusion volume; Vivascience AG).

### 2.2. Crystallization and data collection

The enzyme was crystallized at 296 K by the sitting-drop vapor-diffusion method using 96-well plates (CrystalQuick, Greiner Bio-One) from a solution consisting of 30–34% MPD, 5% PEG 400, 100 m*M* Tris–HCl pH 8.5. The concentration of the protein was 16 mg ml^−1^ in 50 m*M* HEPES pH 7.0. Drops were prepared using 1 µl protein solution mixed with 1 µl reservoir solution and were equilibrated against 100 µl precipitant solution.

Initial crystallization conditions were found using JBSscreen Classic (Jena Bioscience, Germany) and were optimized. Crystals grew in five to six months. The crystals belonged to the primitive monoclinic space group *P*2_1_, with unit-cell parameters *a* = 50.42, *b* = 143.17, *c* = 60.07 Å, β = 107.3°. The asymmetric unit contains one homotetramer (*V*_M_ = 2.21 Å^3^ Da^−1^, 44.42% solvent content).

Data extending to a maximum resolution of 2.1 Å were collected at a temperature of 100 K without using any cryoprotectant on the XRD2 beamline at the Elettra synchrotron, Trieste, Italy at a wavelength of 1.000 Å using a Dectris PILATUS 6M detector. Data processing with *XDS* (Kabsch, 2010 ▸) gave an *R*_merge_ of 16.6% and an overall completeness of 99.9%. The data-collection statistics are summarized in Table 1 ▸.

### 2.3. Structure solution and refinement

The structure was solved by the molecular-replacement technique using *MOLREP* (Vagin & Teplyakov, 2010 ▸) with the coordinates of a theoretical model predicted by the AI program *AlphaFold* from Google DeepMind (Varadi *et al.*, 2022 ▸) as a starting model. Initially the program found a dimer, which was then used for a new *MOLREP* run using the locked rotation function to find the other two subunits.

After the first cycles of refinement using *REFMAC*5 (Murshudov *et al.*, 2011 ▸) from the *CCP*4 package (Agirre *et al.*, 2023 ▸), the model was improved using the *phenix.morph_model* tool (Liebschner *et al.*, 2019 ▸). Refinement was then continued with *REFMAC*5. Water molecules were added automatically with *ARP*/*wARP* (Lamzin & Wilson, 1993 ▸). A model of the enzyme was built using *Coot* (Emsley *et al.*, 2010 ▸). Electron density was missing for residues 180–207 of chain *A*, residues 182–207 of chain *B* and *C* and residues 183–207 of chain *D*. Global structure superpositions were carried out utilizing *SUPERPOSE* (Krissinel & Henrick, 2004 ▸) in *CCP*4.

Ribbon diagrams and other representations were prepared using *CCP*4*mg* (McNicholas *et al.*, 2011 ▸). The coordinates of the protein were deposited in the PDB as entry 8ch4.

Refinement resulted in *R*-factor and *R*_free_ values of 21.29% and 26.12%, respectively. Data-refinement statistics are summarized in Table 1 ▸.

### 2.4. Docking experiments

Docking experiments were performed using the solved crystal structure of 5NSDO as the receptor. The coordinates for the ligand molecules were constructed using *CHEMDRAW* and *Chem*3*D* (version 22.0.0.22, PerkinElmer Informatics). The setup was performed with the *YASARA* molecular-modeling program (version 22.9.24; Krieger & Vriend, 2015 ▸) and molecular-docking experiments were performed using *AutoDock Vina* (Trott & Olson, 2010 ▸) with default parameters, except for the number of runs, which was set to 250. The docking volume consisted of a cubic box with 14 Å edge length centered on the Fe atom of one subunit of 5NSDO. The alanine residues at position 58 in all four sub­units of 5NSDO were replaced with tyrosine rotamers using the Dunbrack rotamer library (Shapovalov & Dunbrack, 2011 ▸) implemented in *UCSF Chimera* (version 1.16; Pettersen *et al.*, 2004 ▸) since the tyrosine side chain was not visible in the electron-density maps. To remove possible bumps and clashes in the generated Tyr58-5NSDO structures, these were subjected to the energy-minimization routine implemented in *YASARA* prior to the docking experiments.

## 3. Results and discussion

### 3.1. Three-dimensional structure of 5NSDO

**Figure 1:**
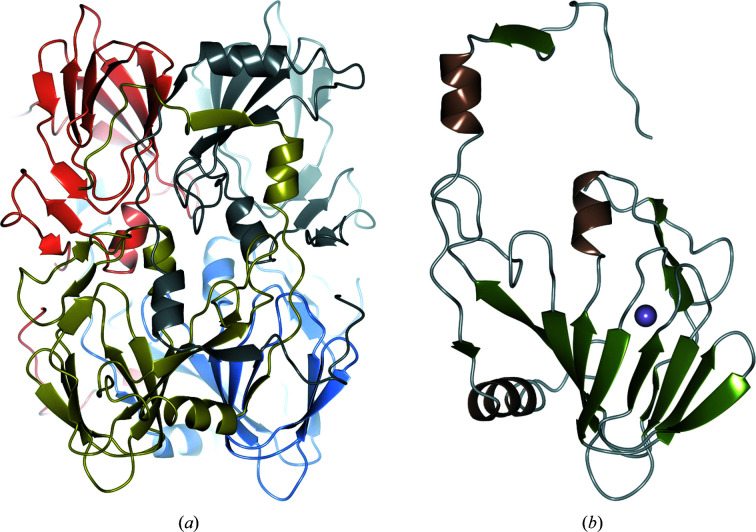
Ribbon diagrams of (*a*) the tetrameric structure of 5NSDO and (*b*) one single subunit. In (*b*) the iron ion is also shown as a violet sphere.

The crystal structure of 5NSDO from *Bradyrhizobium* sp. was determined at 2.1 Å resolution by the molecular-replacement technique using a theoretical model predicted by *AlphaFold*. The asymmetric unit contains one tetramer (see Fig. 1 ▸). Analysis of crystal contacts using *PISA* (Krissinel & Henrick, 2007 ▸) suggested that the tetramer is the biologically active molecule. The four independent copies in the asymmetric unit are very similar (pairwise root-mean-square deviation of 0.22–0.31 Å).

The final model includes a total of 161 water molecules, residues 1–182 of each monomer (molecular mass of about 23.6 kD; the exceptions are reported in Section 3) and one iron(II) ion. The last 25 residues of every subunit of 5NSDO were not modeled due to the poor electron density corresponding to this part of the molecule.

The highest levels of homology of 5NSDO were found to be with GDO from *E. coli* O157:H7 (38.7% identity), 1-hydroxy-2-naphthoate 1,2-dioxygenase from *Nocardioides* sp. (33.1% identity) and SDO (27.5% identity) among dioxygenases of the same class.

**Figure 2:**
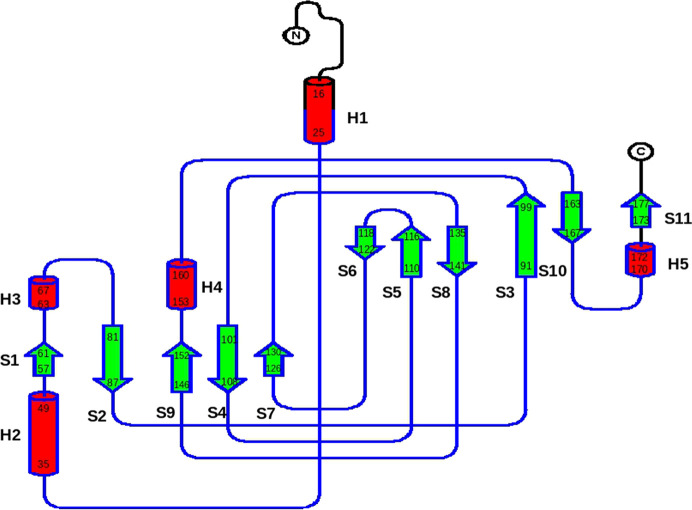
Topology diagram of 5NSDO. The figure was prepared with *TOPDRAW* from the *CCP*4 package. Helices are represented as red cylinders and β-strands as green arrows.

Each subunit of 5NSDO folds as a monocupin domain (residues 81–152) which consists of eight β-strands organized into two four-stranded antiparallel β-sheets that are a characteristic feature of this superfamily (β-barrel). These eight β-strands are additionally flanked by three other β-strands, formed by residues 57–61 (S1 in Fig. 2 ▸) at the N-terminus and residues 163–167 and 173–177 in the C-terminal sequence (S10 and S11 in Fig. 2 ▸). The N-terminus also contains three α-helices, two of which are longer (residues 16–25 and 35–46) and are separated by an unstructured stretch of ten residues (H1 and H2 in Fig. 2 ▸).

Like 5NSDO, other dioxygenases of the same family are also composed of only one cupin motif, for example 3-hydroxyanthranilate 3,4-dioxygenase and 4-amino-3-hydroxybenzoate 2,3-dioxygenase (Fetzner, 2012 ▸). Nevertheless, there are also cupin dioxygenases, such as GDO from *E. coli* and SDO, which are bicupins with two germin-like β-barrel domains. In the bicupins the typical N-terminal β-barrel structural motif comprises the residues that coordinate the catalytic iron ion, whereas the C-terminus is largely mutated at the consensus sequence, so that it lacks the metal-binding residues and remains as a nonfunctional vestigial remnant. There are also examples of bicupins that contain two ferrous centers located in the two homologous cupin domains, such as gentisate 1,2-dioxygenase from *Silicibacter pomeroyi* (Chen *et al.*, 2008 ▸) and quercetin 2,3-dioxygenase from *Bacillus subtilis* (Gopal *et al.*, 2005 ▸). Conversely, hydroquinone 1,2-dioxygenases are heterotetramers constituted of two different subunits each containing a cupin domain, only one of which features a functional metal-binding site (Ferraroni *et al.*, 2017 ▸).

**Figure 3:**
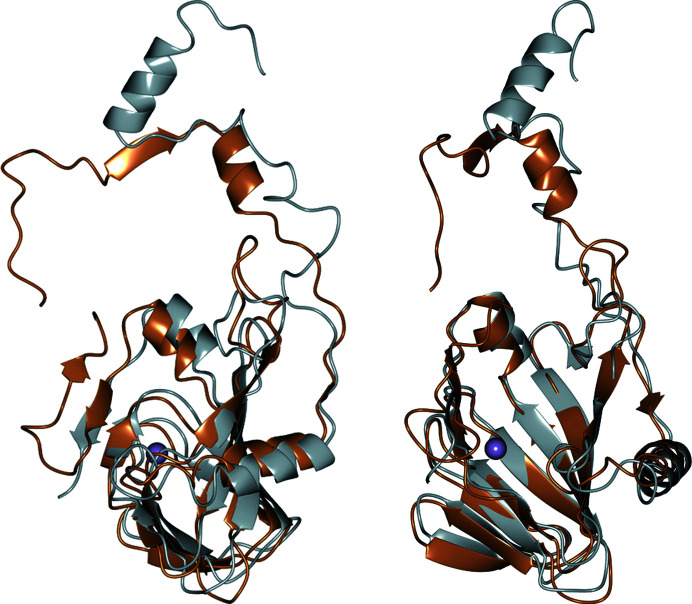
Superposition of one subunit of 5NSDO (gold) with the N-terminal domain of SDO (gray) in two different orientations. The iron ion is represented as a violet sphere.

The 5NSDO structure resembles that of the N-terminal domain of GDO and SDO. A superposition of a single subunit of 5NSDO and the N-terminal domain of SDO is shown in Fig. 3 ▸. The main differences in the SDO structure are in the N-terminal region. The SDO sequence is 20 amino acids longer at the N-terminal end, but the first 16 amino acids of SDO are usually missing in X-ray structures due to inadequate density, probably caused by mobility of this tail. This portion of the polypeptide chain (residues 1–31 of 5NSDO and 17–56 of SDO) is mostly helical in the two enzymes and has a similar conformation, except for the first 15 amino acids, which point towards the β-barrel motif of the same subunit in each subunit of 5NSDO, whereas in SDO they are oriented in the opposite direction, being rotated by almost 180°. Also, the loop connecting helix S2 and strand H1, corresponding to residues 48–57 in 5NSDO, has a different conformation with respect to SDO and the loop connecting strand S1 and strand S2 (residues 62–75) is longer in 5NSDO than in SDO.

The tetramer is mainly stabilized by the long flexible N-terminal region of each subunit, which makes interactions with all three of the other subunits. The four subunits which form the tetramer are related by 222 symmetry, so that the flexible N-terminal peptides are packed in pairs at each face of the tetramer. The resulting oligomeric state results in the formation of a large, square ring structure with a central tunnel. The quaternary structure is arranged in such a way that the active metal sites are oriented towards the external edge of the tetramer.

### 3.2. Active site of 5NSDO and comparison with SDO and GDOs

**Figure 4:**
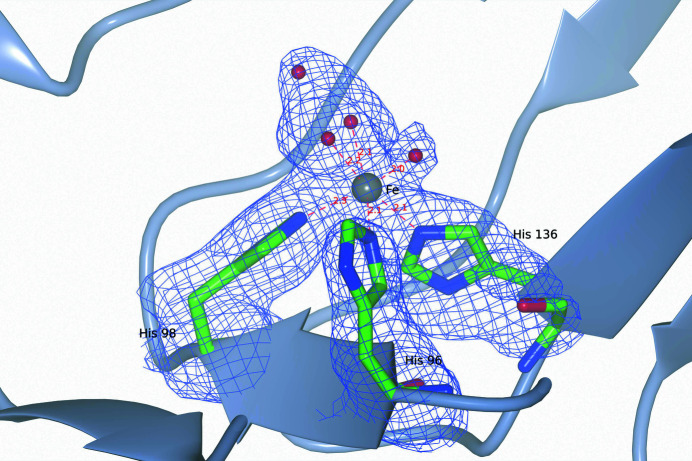
Metal site of 5NSDO showing the iron(II) ion and the coordinating residues. The omit *F*_o_ − *F*_c_ electron-density map is contoured at 2.5σ. The omit map was calculated after 30 refinement cycles with *REFMAC*5, omitting the iron ion, the coordinating histidines and water molecules from the calculation.

The catalytic center contains a mononuclear iron(II) ion bound to three histidines (His96, His98 and His136) and three water molecules with an overall octahedral geometry (see Fig. 4 ▸). Two of the coordinating waters have another water at hydrogen-bond distance, forming a triangular density that could be fitted almost equally well by a carbonate or an acetate ion. The three coordinating histidines belong to two connected β-strands of the β-barrel and are conserved among the cupin dioxygenases.

Cupins are characterized by two motifs, which were originally designated G(*X*)_5_H*X*H(*X*)_3_,_4_E(*X*)_6_G (motif 1) and G(*X*)_5_P*X*G(*X*)_2_H(*X*)_3_N (motif 2), although it is becoming clear that the primary sequence of the two motifs is much less conserved than previously suggested (Dunwell, 2004 ▸). The two histidine residues and the glutamate residue in motif 1, to­gether with the histidine residue in motif 2, can act as ligands for binding the active-site metal. In fact, the cupin dioxygenases can have a 3-His metal coordination site as in SDO (Matera *et al.*, 2008 ▸) and the GDOs (Adams *et al.*, 2006 ▸; Chen *et al.*, 2008 ▸) or a 2-His-1-Glu site as in 3-hydroxy­anthranilate 3,4-dioxygenase (Zhang *et al.*, 2005 ▸) and hydroquinone 1,2-dioxygenase (Ferraroni *et al.*, 2017 ▸) (see also extradiol dioxygen­ases with a 2-His-1-carboxylate facial triad).

Besides the protein ligands, the metal coordination center in the cupin dioxygenases include one, two or three waters or other small ligands as in SDO, where a monodentate carbonate ion was found bound to the iron(II) ion.

**Figure 5:**
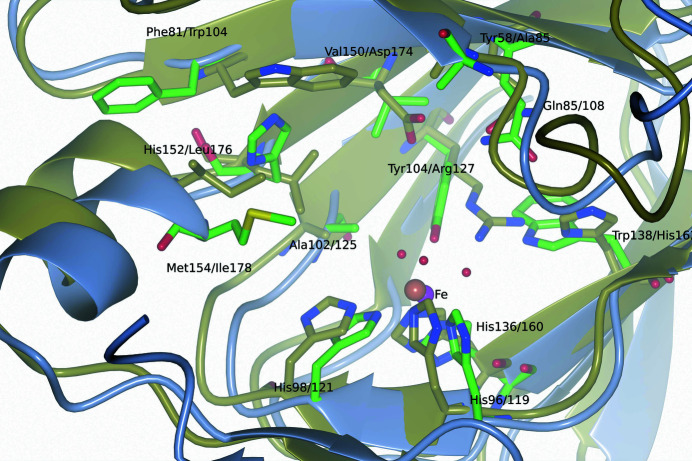
Superposition of the active site of 5NSDO (green) with that of SDO (gold). The iron(II) ions are represented as spheres colored magenta (5NSDO) and orange (SDO).

The active-site residues of 5NSDO are poorly conserved compared with SDO and GDOs. The only identical residues are Arg56, Gly83, Gln85 and Ala102 (Arg83, Gly106, Gln108 and Ala125 in SDO; Fig. 5 ▸).

Arg83 and Gln108 in SDO form hydrogen bonds to the carboxylate O atoms in the structures of complexes of SDO with substrates (salicylate, gentisate and naphthoate; Ferraroni, Steimer *et al.*, 2012 ▸). In particular, upon the binding of the substrates Arg83 moves towards the catalytic cavity through a very pronounced shift of the main chain and a synchronized rotation of its side chain that place this residue in the proximity of the bound substrates, whereas in the unbound SDO structure it is located very far from the active-site iron ion.

In SDO two other residues, Arg127 and His162, make hydrogen bonds to the carboxylate of the substrate. In 5NSDO Arg127 is mutated to a tyrosine (Tyr104) and His162 to a tryptophan (Trp138). Since it has been established that the substrate binds to the ferrous iron in SDO in the deprotonated form (Eppinger *et al.*, 2015 ▸), the function of Arg127 has been identified as essential in order to stabilize the deprotonated hydroxyl group of salicylate (Eppinger *et al.*, 2015 ▸; Roy & Kästner, 2016 ▸). The hydroxy group of salicylate has an extraordinary high p*K*_a_ (13.8) compared with catechol and other substituted phenols, and the presence of the positive charge of arginine in the active site has been related to the necessity of decreasing the p*K*_a_ to favor deprotonation. This was substantiated by mutagenesis studies, which revealed that Arg127 is essential for catalysis (Eppinger *et al.*, 2015 ▸). On the other hand, the phenolic group of 5-nitrosalicylate has a lower p*K*_a_ (9.9; Aydin *et al.*, 1997 ▸) compared with salicylate and in this case the stabilization effect of an arginine is not likely to be necessary for deprotonation.

In the upper part of the catalytic cavity the residues are even less conserved. Ala85, Asp174, Trp104 and Gly106 in SDO are substituted by Tyr58, Val150, Phe81 and Gly83, respectively, in 5NSDO. In SDO, Asp174 and Trp104 are important residues for the binding of gentisate. The inter­actions of Trp104 and Asp174 with the hydroxyl substituent in the 5-position appear to play a central role in substrate recognition. While Asp174 is a highly conserved residue in all known GDOs, residue 104 is a tyrosine in the sequences of most GDOs. In 5NSDO the two residues Asp174 and Trp104 are substituted by Val150 and Phe81, respectively. Besides, the side chain of Phe81 is rotated with respect to the conformation assumed by Trp104 in SDO and is directed towards the opening of the cavity. Hence, in this conformation the side chain of Phe81 would be far from the bound substrate in 5NSDO. These findings may explain the lack of activity of 5NSDO towards gentisate. In fact, as already mentioned, 5NSDO converts 5-nitrosalicylate as well as 5-chlorosalicylate, whereas it is not active towards gentisate.

Tyr58 (Ala85 in SDO) has already been supposed to interact with the nitro group of 5-nitrosalicylate, thus possibly contributing to the observed differences in substrate affinity (Ferraroni, Matera *et al.*, 2012 ▸). Unfortunately, in all four 5NSDO subunits electron density for the side chain of Tyr58 was absent, so it was not included in the model. Nevertheless, on superposing the active site of 5NSDO with that of the structure of SDO bound to gentisate (PDB entry 3nl1) we observed that the side chain of Tyr58 would not have the correct distance and geometry to form a hydrogen-bonding interaction with the substituent in the 5-position of gentisate or 5-nitrosalicylate oriented as gentisate.

Hence, we also docked 5-nitrosalicylate, 5-chlorosalicylate and gentisate into the active site of 5NSDO in order to understand which residues could play a crucial role in determining the selectivity of 5NSDO. The calculations were performed with the substituted salicylates in the deprotonated form and an alanine or a tyrosine at position 58. In the latter case the side chain was modeled as the three most favorable rotamers.

**Figure 6:**
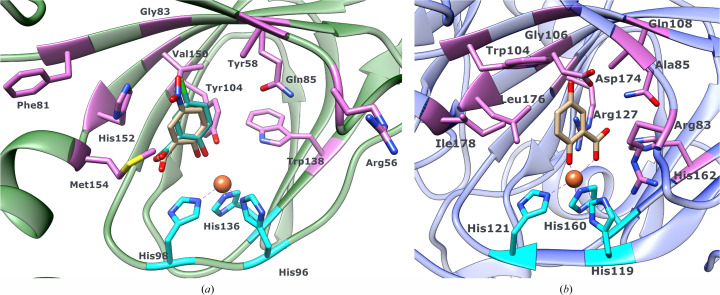
5-Nitrosalicylate (tan) and 5-chlorosalicylate (light green) docked into the active site of 5NSDO (*a*) and the experimentally observed bidentate binding mode of gentisate in SDO (*b*) (PDB entry 3nl1). The histidine residues involved in the coordination of iron(II) are colored cyan. The residues which have been experimentally shown to be involved in substrate coordination and/or discrimination, and that are important for the catalytic activity of SDO, are depicted in purple in (*b*), with the homologous residues in 5NSDO highlighted in the same color in (*a*). Met46 and Leu38 in the N-terminal part of the neighboring subunit in the SDO–gentisate complex (see text) are omitted for clarity.

In the case of docking with an alanine at position 58 we obtained an orientation of the two substrates that differed from that of gentisate in the SDO complex (Fig. 6 ▸). 5-Chlorosalicylate and 5-nitrosalicylate bind to iron in a monodentate mode using the 2-hydroxyl group, whereas the carboxylate group is oriented in the opposite direction with respect to the structure of the SDO–gentisate complex, forming a hydrogen bond with Ser100. On the other hand, gentisate binds in a bidentate way to the iron ion using the carboxylate O atoms, while the 2-hydroxyl group is hydrogen-bonded to Tyr104. The substituents in the 5-position in all three molecules do not make relevant interactions with the residues of the active site.

The results obtained with a tyrosine at position 58 differed depending on the type of substituent at the 5-position of the salicylate and on the conformation of the Tyr58 side chain, but in almost all of the docking poses it was found that the three molecules did not coordinate the metal ion and did not make proper interaction with the Tyr58 side chain. Nevertheless, the stretch of polypeptide chain containing Arg56, the homologue of Arg83 in SDO, has a similar sequence in the two enzymes (Eppinger *et al.*, 2015 ▸) and it cannot be excluded that it moves upon substrate binding as in SDO (see above), also inducing a conformational change in Tyr58 which could trigger more suitable interactions with the substrate.

His152 and Met154 (Leu176 and Ile178, respectively, in SDO) also form part of the active site of 5NSDO. The side chain of His152 could make a π-stacking interaction with the aromatic ring of the substrate, stabilizing its binding inside the catalytic cavity in a similar mode to Trp104 in SDO.

In SDO the active site is composed of residues belonging to one subunit of the tetramer and residues of the N-terminal end of another subunit, for example Met46 and Leu38. In 5NSDO the different conformation and amino-acid composition of the N-terminal peptide (for example Leu38 is replaced by Ala17 in 5NSDO) reduces its involvement in the formation of the active site and its ability to influence the substrate selectivity of the enzyme.

### 3.3. Comparison with the *AlphaFold* model

*AlphaFold*2 was made publicly available in 2021 (Varadi *et al.*, 2022 ▸; Jumper *et al.*, 2021 ▸). The impressive results produced by *AlphaFold*2 at the CASP14 (Critical Assessment of Techniques for Protein Structure Prediction) contest indicated that deep learning-based methods are now able to predict protein structures with an accuracy comparable, in most cases, to that of experimental structures (Pereira *et al.*, 2021 ▸).

The structure of 5NSDO was solved quite straightforwardly by molecular replacement using an *AlphaFold*2 model. Previous attempts to solve the structure using models built from the coordinates of homologous cupin dioxygenases were unsuccessful, confirming the high accuracy of the *AlphaFold* model. This fact underlines the importance of *AlphaFold* for solving the phase problem in X-ray protein crystallography, as reported in recent studies (Millán *et al.*, 2021 ▸). McCoy and coworkers explored the impact of *AlphaFold* models on phasing by molecular replacement (McCoy *et al.*, 2022 ▸). Using the *in silico* model provided by *AlphaFold*2 for 34 targets submitted to the CASP14 contest, they were able to solve the structures of 31 of these proteins using molecular replacement. The *AlphaFold*2 model has also been used to solve the SARS-CoV-2 ORF8 structure retrospectively by molecular replacement (Flower & Hurley, 2021 ▸). Furthermore, the structure of Nmd4 from *Saccharomyces cerevisiae*, a protein involved in the nonsense-mediated mRNA decay pathway, has been solved by molecular replacement using models generated by *AlphaFold*2 after extensive efforts to try to solve the structure by the methods commonly used in macromolecular X-ray crystallography (*i.e.* MIR, MAD/SAD and molecular replace­ment).

**Figure 7:**
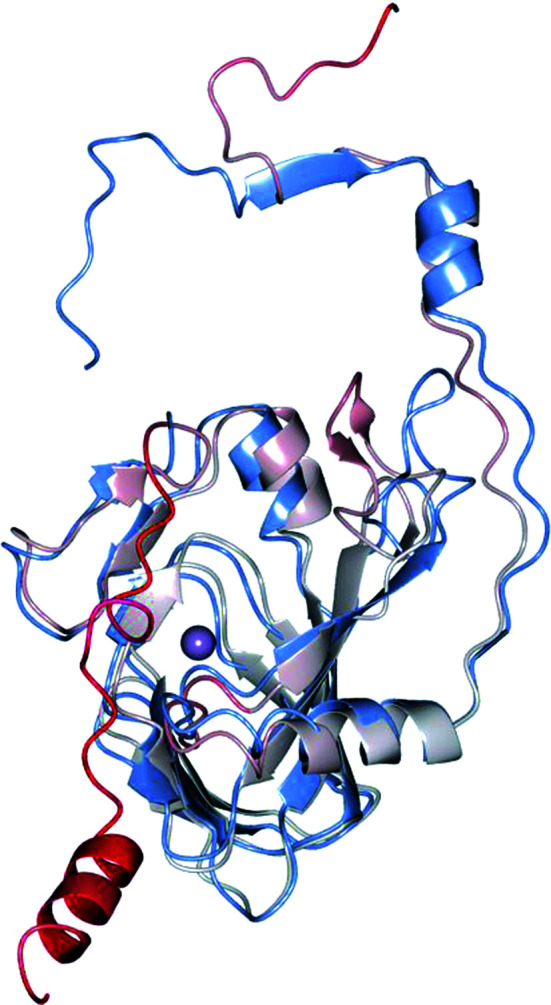
Superposition of one subunit of 5NSDO (light blue) with the *AlphaFold* model used for phasing by molecular replacement colored by per-residue confidence estimate pLDDT (a red color means low confidence and gray very high confidence). The iron ion is represented as a violet sphere. The last 25 residues are absent in the crystallographic structure of 5NSDO.

Comparing the 5NSDO structure with the *AlphaFold* model that was used for phasing by molecular replacement (Fig. 7 ▸), we found that the main differences are in the N-terminal region (the first 14 amino acids point in different directions) and at loop 62–75, which has a similar conformation in the two models but is slightly rotated in the direction of the β-barrel in the *AlphaFold* model. The final crystal structure and the *AlphaFold* model superpose with an r.m.s.d. of 5.15 Å over 180 C^α^ atoms. Nevertheless, a comparison of the active sites between the experimental model and the AI model reveals differences in the conformations of a number of amino-acid side chains that could affect an accurate analysis of the factors contributing to the enzyme mechanism and substrate selectivity. Furthermore, the *AlphaFold* model does not contain information about the quaternary structure of the enzyme and the positions of the metal ions, cofactors and other small molecules which are important for detailed knowledge of enzyme function.

## 4. Conclusions

The X-ray structure of 5NSDO from *Bradyrhizobium* sp., solved by molecular replacement using a theoretical *AlphaFold* model, shows that the enzyme is a tetramer of identical monocupin subunits. In the active site the iron(II) ion is coordinated to three histidines, the most typical coordination motif for cupin dioxygenases.

Comparing the 5NSDO structure with those of closely related ring-cleaving dioxygenases of the same class, for example SDO and GDO, we found that the active-site residues are poorly conserved, as expected from the unusual activity exhibited by the enzyme, which accepts 5-chloro­salicylate and 5-nitrosalicylate as substrates but not gentisate and salicylate. In fact, many of the residues that have been identified as crucial for catalytic activity and substrate recognition in SDO and GDOs are mutated in 5NSDO. These substitutions in the active site of 5NSDO described in the discussion are responsible for the very different selectivity of 5NSDO with respect to that of GDOs and SDO. Specifically, the residues which interact with the 5-OH group of gentisate (Asp174 and Trp104) are missing in 5NSDO, explaining the lack of activity of 5NSDO towards gentisate. Arg127, which is supposed to facilitate the deprotonation of the substrate in SDO, is substituted by Tyr104 in 5NSDO, likely due to the lower p*K*_a_ of the substrate hydroxyl group compared with salicylate. The residue at position 58 was supposed to be particularly important for establishing favorable interactions with the nitro group in the 5-position. The lack of electron density for the side chain of Tyr58 did not allow proper identification of the interaction of this residue with the substrate, even when docking 5-nitrosalicylate into the active site of 5NSDO.

The next step will be to confirm these findings by analysis of the catalytic properties of 5NSDO mutants and by determining the structures of enzyme–substrate complexes of the wild-type enzyme and mutants.

## Supplementary Material

PDB reference: 5-nitrosalicylate 1,2-dioxygenase, 8ch4

## Figures and Tables

**Table 1:** Data-collection and refinement statistics Values in parentheses are for the highest resolution shell.

Data collection	
Temperature (K)	100
Space group	*P*2_1_
*a*, *b*, *c* (Å)	50.42, 143.17, 60.07
α, β, γ (°)	90.0, 107.3, 90.0
Resolution (Å)	48.14–2.10 (2.23–2.10)
Unique reflections	46889 (7465)
*R*_merge_ (%)	16.6 (158.7)
*R*_meas_ (%)	18.0 (172.2)
〈*I*/σ(*I*)〉	9.09 (1.35)
Completeness (%)	99.9 (98.4)
Multiplicity	6.8 (6.6)
CC_1/2_	99.7 (68.2)
Refinement	
Resolution (Å)	48.14–2.10
No. of reflections (*R*_work_/*R*_free_)	44636/2202
*R* factor (%)	21.29
*R*_free_ (%)	26.12
*B* factors (Å^2^)	
Protein	46.49
Ions	33.13
Waters	40.97
Ramachandran statistics (%)	
Most favored	98.3
Additionally allowed	1.7
Outlier regions	0.0
R.m.s. deviations	
Bond lengths (Å)	0.0062
Bond angles (°)	1.432
PDB code	8ch4
